# Stakeholder Attitudes Toward the Implementation of School-Based, Universal, Mental Health Screening: Student, Caregiver, and Teacher Perspectives

**DOI:** 10.3390/ijerph22121825

**Published:** 2025-12-05

**Authors:** Ronald M. Rapee, Rebecca-Lee Kuhnert, Ian Bowsher, John Burns, Julie Dixon, Catherine Lourey, Lauren F. McLellan, Traci Prendergast, Viviana Wuthrich

**Affiliations:** 1 Lifespan Health and Wellbeing Research Centre, School of Psychological Sciences, Macquarie University, Sydney, NSW 2109, Australia; rebecca.kuhnert@mq.edu.au (R.-L.K.); john.burns@mq.edu.au (J.B.); lauren.mclellan@mq.edu.au (L.F.M.); viviana.wuthrich@mq.edu.au (V.W.); 2 Sydney Secondary College, Rozelle, NSW 2039, Australia; 3 The Mental Health Commission of NSW, Sydney, NSW 2001, Australia; 4 Psychology and Wellbeing Services, NSW Department of Education, Sydney, NSW 2001, Australia

**Keywords:** universal screening, mental health, school, assessment, service delivery

## Abstract

This paper reports on data from two trials about stakeholders’ attitudes to school-based mental health screening. Study 1 reports data from 6228 students from grades 4 to 12 while Study 2 reports data from 267 caregivers and 34 educators from a larger trial. All three groups of stakeholders reported broadly positive attitudes toward school-based screening. Few students reported distress from questions and most agreed that schools should screen. Caregivers and educators reported positive attitudes toward the use and implementation of screening and reported few concerns about harms. Educators who conducted screening reported mostly positive experiences, although they noted high resource burden and false positives and negatives.

## 1. Introduction

Universal mental health screening in schools has received growing attention as one strategy to promote early identification of at-risk youth [1,2]. Unfortunately, the uptake of screening remains low around the world [3,4,5]. In one survey of school psychologists we found that concerns about the acceptability of screening by school staff, caregivers, and students was a major barrier to its adoption [4].

Research has begun to evaluate the attitudes of relevant stakeholders to school-based screening. Primary focus has been on school staff, with most surveys showing broadly positive views on the concept of screening, although with practical concerns for its implementation. For example, a survey of pre-school teachers identified 73% indicating that screening was ‘useful’ and 65% that screening was ‘important’ [6]. However, only 56% said that screening whole classes was ‘feasible’. Another study of 40 pre- and elementary teachers, showed that most ‘agreed’ or ‘strongly agreed’ that surveys were useful to identify students with difficulties, but their endorsement of the feasibility of screening and their willingness to complete screeners was more commonly in the ‘slightly agree’ to ‘agree’ range [7]. A qualitative study of primary school teachers’ views on universal screening supported a broadly positive view, although concerns were expressed about resource requirements; potential psychiatric labelling of students; and the possibility of false negative or positive results [8].

Teacher acceptability of screening appears to be moderated by the problem being screened for. For example, a systematic review on the feasibility of school-based identification approaches found that teachers were more likely to accept screening if the focus directly matches school priorities such as behavioural and socioemotional difficulties or eating disorders [9]. In contrast, screening for suicidal ideation has received less support from school personnel [10]. A recent systematic review into the acceptability of universal mental health screening found that school staff were generally favourable towards screening particularly once they had an experience of screening, with scores collected concurrently or post screening (*M* = 4.16, *SD* = 0.59) being higher than in those studies carried out prospectively (*M* = 3.33, *SD* = 0.58) [11]. In their analysis of qualitative studies, Palmer and colleagues reported school concerns about the burden placed on staff as well as issues of confidentiality and risk of labelling.

Relative to attitudes by school staff, less research has evaluated the attitudes of parents or caregivers toward school-based mental health screening. Nonetheless, identified attitudes have broadly been positive. For example, in a study of 254 parents of primary school children in the UK, 82% of parents thought that screening should be helpful and only 13% were concerned about potential harms [12]. Similarly, in a very large survey of parents from China (*N* = 13,480), most (92%) reported a “willingness to accept” school-based screening for depression [13]. However, more than one third expressed concerns about the screening worsening symptoms of depression. Those who did not endorse screening were found to have less accurate knowledge about depression and held more stigmatising beliefs about depression and suicide. In their study of 770 parents of middle/high school students in the US, Sekhar and colleagues [14] found most parents support screening for depression in schools, but that a sizeable minority (15.9%) held objections. In another study with parents of pre- and elementary school students, parents strongly endorsed school screening [7]. Using a 5-point scale, parents agreed that it is important for schools to ask questions about children’s emotions/behaviour (*M* = 4.60) and that surveys about children’s emotions and behaviour are useful in identifying which students need help (*M* = 4.49) [7]. A very large study across 1330 American school districts found that 58–76% of parents believed that schools should definitely check students’ mental health and that universal screening was the best method to do so [15]. Parental acceptance also extends to screening students for self-harm and suicidal ideation, with more than 85% of parents expressing support [16,17]. Despite a general theme of acceptance from parents for school-based universal screening, the literature on parent attitudes is limited both in terms of number and scope. In the few studies that have been done, many focus on depression and/or suicide screening, with comparatively fewer on screening for general distress or other disorders.

Even less is known about students’ views on universal school-based mental health screening, a surprising limitation given that students are arguably the core focus. One study with 272, 14–16 year-old boys who completed a questionnaire on self-harm and suicidal ideation found that 81% did not find the screening questions intrusive and 72% described it as worthwhile [17]. A New Zealand study of 21, grade 9 students found that they mostly agreed that the online format “works for people my age” (85.7%), although almost one quarter (23.8%) said that the “questions are too personal”, and 42.9% “worried about the privacy of my information” [18]. A small qualitative study in the UK interviewed 51 grade 9 students after completing a broad, online health and wellbeing screening programme, including some items on mental health [19]. The screening was primarily conducted when the students were at home during COVID lockdown. Most respondents reported the process to be helpful, and found both the on-line nature of the screening, as well as the alternative of completing the screener at home rather than at school allowed for more privacy and consideration of their responses. Clearly, data on students’ attitudes to screening comes from a very small number of small studies focussing on specific contexts.

Overall, there is a growing, but still limited empirical literature on the attitudes of stakeholders to the use of school-based, mental health screening. This literature shows general acceptability of school-based screening and little concern for potential harms. However, several studies focused on screening for specific issues such as depression or suicide or restricted their study to a limited range of students. Very few studies compared perspectives between stakeholders and there is currently little research evaluating caregivers’ attitudes and minimal research evaluating the attitudes of perhaps the primary stakeholders, students.

The current studies aimed to evaluate the attitudes toward school-based mental health screening across three, primary stakeholder populations, albeit in two, separate screening contexts—Study 1 assessed students’ perspectives while Study 2 reports on caregiver and clinician perspectives. Screening covered a range of common emotional difficulties and included students from grades 4 to 12 across a broad variety of schools. Based on previous evidence, we expected generally positive attitudes and expected more positive attitudes from school staff than from caregivers [7]. We did not hold specific hypotheses about students’ perspectives, given the very limited prior evidence base. We also asked school staff who had been involved in prior screening about their intentions toward future screening, although in the absence of previous evidence, we held no specific hypotheses.

## 2. Method—Study 1

### 2.1. Participants

Participants for Study 1 comprised 6228 students from grades 4 through 12 from 15 Government (general public) schools in Sydney, Australia. Schools volunteered their participation. There were 3111 students who identified as male (50.0%), 3086 identified as female (49.6%), and 25 identified as “Indeterminate, intersex, or unspecified” (0.4%). Mean age was 11.84 years (*SD* = 2.26), ranging from 7 years to 18 years. Additional demographic data were not collected, but the schools were all located in middle to upper-middle income areas of Sydney with a primarily Caucasian population.

### 2.2. Measures

Measures of mental health included symptoms of anxiety (Spence Children’s Anxiety Scale) [20]; depression (Short Mood and Feelings Questionnaire) [21]; body image (Body Esteem Scale for Adolescents and Adults, appearance and weight subscales) [22], and victimisation (Olweus Bully Victim Questionnaire, vicitmisation subscale) [23]. Students in high school (grades 7 and above) were also asked a few questions developed for this study about intent toward self-harm or suicide.

At the completion of the mental health and experimental measures, students responded to the following five questions (questions for this and the next study were developed by the authors based on personal experience and were not derived from existing measures; see Table 1):

1. Did you get upset when you were answering any of the questions in this survey: 1 (not at all) to 5 (extremely upset)?
2. If students answered greater than 1 to the above question, they received an open-ended question asking which items made them feel distressed.
3. How important do you think it is for kids to know about emotional health: 1 (not at all important) to 5 (extremely important)?
4. Do you think schools should check whether students have emotional health worries: 1 (definitely not), 3 (no opinion), 5 (Definitely yes)?
5. Do you think schools should teach students about emotional health: 1 (definitely not), 3 (no opinion), 5 (Definitely yes)?

### 2.3. Procedure

The 15 schools in this study initiated contact with the first author (RR) to conduct school-based mental health screening. Opt-in consent process was used, providing information to caregivers via usual school processes (e.g., newsletter) followed by emailing consent forms to all caregivers that had to be returned for their child to be included. The total number of distributed consents was not counted and therefore the exact engagement rate cannot be determined. However, most schools reported around 60% of their students returned opt-in consent. Once caregivers had provided consent, students were also asked for their consent prior to commencing the survey.

Students completed surveys in school time (approx. 30 min), in class groups, and were supervised by a teacher. A school counsellor/psychologist was always available during testing times if required. Surveys were conducted through Qualtrics and the data (identified only by student number) was available to the primary researcher (RR). Schools were provided feedback and support about any student who scored above cut-off. The overall study was approved by the Macquarie University Human Research Ethics Committee.

## 3. Method—Study 2

### 3.1. Participants

Participants for Study 2 were caregivers and educators involved in a randomised controlled trial of the efficacy of school-based mental health screening and feedback [24]. There were 267 caregivers and 34 educators who opted in to providing data. Caregivers primarily identified as female (*n* = 236, 88.4%) and the biological parent of the student (*n* = 260, 97.4%). Their children identified as male (*n* = 145, 61.7%), female (*n* = 84, 31.5%), or other (*n* = 6, 2.2%; 32 missing), with a mean age of 11.85 years (*SD* = 1.99), from grades 4 to 11 and attended Government (general public) (*n* = 195, 73.0%) and non-Government (Catholic or independent/private) (*n* = 72, 27.0%) schools.

Of the 34 educators, 29 (85.3%) identified as female and their roles in the schools included: counsellor/psychologist (*n* = 9, 35.3%); specialist teacher (*n* = 6, 17.6%); school principal (*n* = 9, 26.5%); or other (including classroom teacher or chaplain: *n* = 7, 20.6%). The sample had worked in the school system for an average of 16.86 years (*SD* = 12.03; range, 0.75 to 36 years). They came from both Government (*n* = 27) and non-Government (*n* = 7) sectors.

### 3.2. Measures

Students in Study 2 completed mental health measures including the Brief Evaluation of Adolescents and Children Online (BEACON), which includes items about symptoms and associated impairment for anxiety, depression, attention/activity, and eating difficulties [25]. Caregivers who volunteered were asked about their attitudes to school-based mental health screening. This measure included 10 items, each utilising a 5-point Likert response scale from 1 (strongly disagree) to 5 (strongly agree), with an additional option for “don’t know”. Items included general questions about the role of schools along with more specific questions about school screening (see Table 2). The 10 items were averaged, with negatively worded items being reversed, so that higher mean scores indicated a more positive caregiver attitude toward mental health screening.

Educators completed slightly different questions depending on whether their school was allocated to conduct screening and feedback at Time 1 or Time 2 (see Procedure). All educators (both groups) completed a 10-item general attitudes measure that was similar to the one completed by caregivers but reworded to fit with the educator perspective. All educators were also asked four additional questions about their attitudes to conducting school-based screening within their school, which are described in Table 2. These latter items utilised four-point Likert scales from not at all true (1) to very true (4).

In addition to these broader questions about mental health screening, only those educators whose schools conducted screening and feedback at Time 1 were asked additional questions about their experiences, answered on 4, or 5-point Likert scales (see Table 3).

### 3.3. Procedure

The larger trial of school-based mental health screening and feedback included 10,660 students from 53 schools across the state of NSW, Australia who completed a set of mental health measures and their caregivers received feedback and support via the schools. Detailed methods and results are reported elsewhere [24]. Caregivers who opted in to the study were sent a broader set of measures (including the above) at a similar time to which their child completed measures in school and prior to receipt of any feedback and support about their child.

Educators who were invited to participate were the primary contact in each school for the larger trial (maximum *N* = 53; 34 respondents = 64%). The larger trial was a randomised controlled trial in which schools were randomly allocated to conduct the screening and feedback at Time 1 (2020) and again at Time 2 (2021) or to only conduct the screening at Time 2 (2021). Educators were invited to complete the attitude measures after their school had conducted the screening, although educators in the Time 2 only condition (*n* = 23) were not asked about their experiences with the screening. The study received primary approval from the Macquarie University Human Research Ethics Committee.

## 4. Results—Study 1

Students’ responses to the five questions are described in Table 1 (divided by grade purely for descriptive purposes). When asked whether any questions upset them, 4321 students (70.9%) responded “not at all”; 1303 students (21.4%) responded “slightly”’ and the remaining 468 students (7.7%) reported moderate or greater distress. However, among students who reported moderate distress or greater, 96 (20.5%) did not describe any specific questions that distressed them and 117 (24.9%) referred to non-mental health aspects of the survey or provided nonsense responses. The specific sources of distress among the remaining 276 students (i.e., those who a; reported that an item made them upset and b; specifically described a mental health item in the open-ended question) (4.5% of the total sample) are shown in Table 1. As can be seen, the greatest identified sources of distress were suicide/self harm items, body image items, and bullying items. As shown in Table 1, students reported very positively on the remaining three questions. Almost all students felt that it is moderately or more important to know about emotional health and over three quarters felt that schools should be involved in assisting students with emotional health.

We explored whether students’ agreement that “schools should check students’ emotional health” was related to demographic or mental health characteristics. There were mostly significant but very small positive correlations between agreeing that schools should check emotional health and student age (*r* = 0.072, *n* = 6010, *p* < 0.001); anxiety (*r* = 0.064, *n* = 6020, *p* < 0.001); depression (*r* = −0.014, *n* = 3344, *p* = 0.405); body image (*r* = 0.042, *n* = 6017, *p* < 0.001); and victimisation (*r* = 0.041, *n* = 3546, *p* = 0.013). Girls (*M* = 4.17, *SD* = 0.90) also reported significantly greater agreement with the statement than boys (*M* = 4.02, *SD* = 0.100), *t*(5995) = 6.13, *p* < 0.001 (insufficient non-binary genders for analysis).

## 5. Results—Study 2

### 5.1. Caregivers

Caregivers mostly agreed with positive statements about mental health screening and disagreed with negative statements (Table 2). The least positive support related to whether young people could accurately report on their mental health, with 25% of caregivers being uncertain or disagreeing. Importantly, around 90% of caregivers did not agree that school-based screening could negatively impact their child.

Associations were examined between demographic descriptors and average scores across the 10 items of caregiver attitudes. There were no significant correlations between average attitude score and either the student’s age, *r* = −0.06, *p* = 0.334, or the student’s mental health (BEACON total score), *r* = 0.004, *p* = 0.952. Similarly, no significant differences were shown between male (*M* = 4.19, *SD* = 0.34) and female (*M* = 4.33, *SD* = 0.40) caregivers, *F*(1, 265) = 3.43, *p* = 0.065, *η*_p_^2^ = 0.013, nor between caregivers whose child attended government (*M* = 4.31, *SD* = 0.42) or non-government (*M* = 4.32, *SD* = 0.32) schools, *F*(1, 265) = 0.10, *p* = 0.921, *η*_p_^2^ < 0.001.

### 5.2. Educators

Educators’ attitudes toward school-based mental health screening were broadly positive (Table 2). All or almost all educators agreed with statements about the importance and value of schools in assessing students’ mental health and disagreed with statements suggesting that screening could result in harms. Educators were less consistent in their agreement that students were capable of honestly responding to items or that all parents should be provided with feedback. Educators also responded positively to questions about their comfort and knowledge in conducting school-based mental health screening and that the community supported this process. Almost 90% indicated that they planned to conduct future screening.

A subgroup of educators (*n* = 11) reported on their experiences conducting school-based mental health screening and follow-up over the previous 12 months (Table 3). Almost all educators reported a generally positive experience with both screening and feedback. With respect to screening, a proportion of educators reported some concerns about whether students could understand the items or whether they took the screening seriously. Educators also noted some negativity around the time required for screening. However, almost no educators reported student distress in response to screening. Regarding provision of feedback, a moderate proportion of educators noted issues around false positives and negatives, although almost all educators believed that the screener successfully identified previously unknown students. Almost half the educators noted the lack of appropriate external referral services. However, most educators believed that caregivers were positive toward feedback and there was universal agreement that the provided guidelines were helpful.

## 6. Discussion

A core barrier to uptake of universal mental health screening by schools is a concern that screening will not be accepted by school communities (including teachers, students, and caregivers) [4]. Contrary to this concern and consistent with previous research [9,11], our results showed general positivity toward the use of both screening questions and the overall screening and feedback process by all stakeholders.

In Study 1, students reported strong agreement that mental health was an important issue for schools to teach students and over three quarters of the students agreed that schools should check on their students’ mental health. These data provide by far the largest and broadest sample in the literature indicating that the primary target stakeholders, students, mostly believe that school-based mental health screening is a worthwhile activity. It suggests that most students will buy into such activities by schools and are likely to provide honest responses. Of course, almost 23% of students did not believe that schools should check students’ mental health, suggesting that school-based screening is not a panacea for all students and that additional methods of mental health promotion are needed. Importantly, very few students (less than 8%) reported significant distress in response to questions (in fact less than 5% referred to a mental health question as distressing), including questions about self-harm and suicidal ideation, loneliness, depression, and poor body image. Concerns that asking about mental health will cause distress is a common issue raised by ethics committees and may be raised by school administration when considering the uptake of mental health screening. These results should help allay those concerns and assist service providers when discussing mental health screening with schools.

None of the student demographic or mental health predictors that we explored demonstrated strong relationships with acceptance of school screening, although it was heartening to see that students reporting greater emotional distress were no less accepting and, if anything, were slightly more accepting of school screening. There were few age or gender differences, although older students and girls were very slightly more accepting of screening.

Study 2 extended these results to caregivers and educators who largely reflected students’ positive attitudes, with 86% of caregivers and 97% of educators agreeing that schools should engage in mental health screening. On the converse, 91% of caregivers and 100% of educators disagreed that mental health screening would be harmful to students. These data are consistent with previous findings pointing to the broad acceptance of school-based mental health screening among caregivers and educators [12,13,26]. Of course our results were conducted within a single country, with relatively small cultural variation and so it is possible that these results will not generalise across cultures and different school systems.

The smaller subset of educators who had completed an engagement with screening, provided important information about their experiences. Positively, there was overwhelming support for the experience of both screening (92%) and following up with identified students (100%). Consistent with the results from students, educators also reported very few students (9%) who expressed any distress during screening. However, they did highlight some concerns: false positives and negatives; significant burden of conducting screening; and limited external services for referral of identified students. These are important issues that schools need to weigh up when deciding whether to engage in mental health screening. Clearly there are time and administrative burdens involved in conducting screening and schools need to ensure they have the motivation, top-down support, and resources to engage in the activity. False positives and negatives are also an inevitable aspect of any screening [27] and schools can mitigate any harms by ensuring that caregivers, students, and staff view the screening as a chance for feedback and further conversation rather than seeing it as a diagnosis or “fait accompli”.

The primary limitation for this research was sampling. Study 1 utilised a large sample of students, which was a major strength, but relied on opt-in consent, with only 60% of the school population being eligible. It is known that opt-in consent biases against certain groups such as lower SES and people with mental health concerns [28,29] and therefore, our results cannot be said to represent the full population. This limitation was even more relevant to the results from caregivers and educators, given the small samples involved. In both cases respondents opted into the study and it is possible that those who volunteered were also more positively disposed toward mental health in general and mental health screening more specifically than those who did not participate. In addition, schools within this study were mostly from middle income areas or above and the majority of participants identified as Caucasian. Hence the largely positive attitudes toward screening expressed in these results need to be accepted cautiously until more broadly representative research is conducted.

Attitudes also do not necessarily translate into actual behaviour. Therefore, it is possible that the highly positive attitudes identified in this research may not have been the same if actual implementation of school-based screening was required. Future studies would benefit from a method in which future implementation is incorporated as an additional indicator of acceptability.

## 7. Conclusions

Research has shown that school-based screening combined with clear feedback guidelines can reduce self-reported mental health symptoms and improve school attendance [24]. It is therefore one method that can help reduce the gap between mental health concerns and appropriate help among young people [30,31]. The current data from two studies shows that school-based mental health screening is broadly acceptable to students, caregivers, and educators, although educators also noted some potential barriers. These results should help schools to plan for screening their student populations in the most acceptable ways. It should also be noted that the current research in addition to the broader research literature is largely restricted to middle socio-economic populations from mostly Western countries and with little to no comparison between differing school systems or communities. Therefore, future research that broadens the research base to encompass far greater diversity would be warranted.

## Figures and Tables

**Table 1:** Responses to survey questions by students (N = 6228).

Question	Percent Agreeing Full Sample	Percent Agreeing Grades 4–6 (n = 3695)	Percent Agreeing Grades 7+ (n = 2394)
Did you get upset when you were answering any of the questions in this whole survey?	7.7% ^1^	6.1%	10.2%
Which questions made you feel upset? (n = 276)			
Anxiety	4.5%	7.3%	3.4%
Depression	4.3%	3.4%	7.3%
Body image	13.9%	17.1%	16.8%
Suicide/self harm ^4^	15.4%	9.3%	29.6%
Victimisation/peer relations	12.8%	25.9%	3.9%
All/everything	3.6%	3.4%	5.6%
How important do you think it is for kids to know about emotional health?	93.4% ^2^	93.1%	93.9%
Do you think schools should check whether students have emotional health worries?	77.2% ^3^	75.8%	79.3%
Do you think schools should teach students about emotional health?	76.9% ^3^	73.8%	81.8%

Note: 1. Percent agreeing coded as: 1. “moderately upset” or higher; 2. “moderately important” or higher; 3. “probably yes” or “definitely yes”. 4. Suicide/self harm questions for grades 4–6 were only reported by students in grade 6. Data broken down by the full set of response options are presented in the Supplementary Material (Table S1).

**Table 2:** Responses to attitude questions by caregivers (*N* = 267) and educators (*N* = 34).

Question	Percent Agreeing (Caregivers)	Percent Agreeing (Educators)
Schools have an important role in making sure that children have good mental health	95.9 ^1^	100.0 ^1^
Schools are well placed to spot the early signs that a child may be experiencing mental health difficulties	86.1	88.3
It is important to identify children experiencing mental health problems as early as possible	99.6	100.0
It is important for schools to carry out mental health checks (screening) for their students	86.2	96.9
Conducting mental health checks (screening) in schools could be harmful to students	8.9	0.0
If a school asks questions about mental health from students, it could put negative ideas in their head	11.2	8.8
I would be prepared to complete a questionnaire about (my child’s/each of my students’) mental health for the purpose of a routine mental health check (screening)	96.5	70.0
(My child/Students at my school) is perfectly capable of honestly and accurately reporting his or her mental health	75.1	33.3
All parents should be provided with feedback about their child’s mental health following his/her participation in a mental health check (regardless of whether they are identified as having difficulties or not)	90.2	54.5
I would be happy to work with (my child’s school/students’ families) and other organisations if a mental health check showed that (my child/student) could benefit from extra support	99.7	94.1
I plan to screen students in my school for mental health concerns in the future	N/A	87.9 ^2^
I feel comfortable knowing how to conduct screening	N/A	82.4
I feel comfortable knowing how to handle students identified with a mental health problem	N/A	91.2
I am confident that the school community supports the idea of mental health screening	N/A	91.2

Notes: Words in parentheses are alternatives for the caregiver and educator versions. Agreement refers to responses coded: 1. “agree” or “strongly agree”; 2. “somewhat true” or “very true”. Data broken down by the full set of response options are presented in the Supplementary Material (Table S2).

**Table 3:** Responses to questions about educators’ experiences with the screening and feedback (*n* = 11).

Question	Percent Agreeing ^1^
*Screening*	
Overall, what was your experience with screening?	90.9 ^1^
Students had trouble understanding the screening questions	27.3 ^2^
Students did not take screening seriously	36.4 ^2^
Students became distressed during/after screening	9.1 ^2^
Screening was difficult to fit into the regular class timetable/schedule	36.4 ^2^
Preparing for and/or conducting screening took up too much administrative time	45.5 ^2^
*Feedback*	
Overall, what was your experience with following up with students who were identified through screening?	100.0 ^1^
The screener worked well to identify students who needed help and were not previously known to school staff as needing help	90.9 ^2^
Too many students were identified who, when followed up, are not experiencing MH difficulties	27.3 ^2^
I know of a number of students who do have mental health difficulties but were not identified through screening	36.4 ^2^
Caregivers for identified students appreciated our contact and the information we provided	81.8 ^2^
Caregivers supported the idea of getting their child additional mental health help when contacted by us	81.8 ^2^
We did not have sufficient time/capacity to follow up identified students	18.2 ^2^
There were not enough external services/resources available to support identified students	45.5 ^2^
The guideline that was provided to us was helpful in providing guidance on how to follow up with identified students	100.0 ^2^

Note: Agreement refers to educators responding: 1. ”quite positive” or “very positive”; 2. “somewhat like my experience” or “very like my experience”. Data broken down by the full set of response options are presented in the Supplementary Material (Table S3).

## Data Availability

Data provided for Study 1 was obtained from a service delivery project and participants did not provide consent for public dissemination of the data. De-identified data can be obtained from the first author. The data provided for Study 2 is part of a larger research project as noted in the methods and data availability is as described in the primary outcome paper.

## Supplementary Materials

The following supporting information can be downloaded at: https://www.mdpi.com/article/10.3390/ijerph22121825/s1, Table S1: Attitudes to screening by students in study 1 describing all levels of response. Table S2: Attitudes to screening by educators and caregivers in study 2 describing all levels of response. Table S3: Feedback about screening by educators who implemented school-based mental health screening in study 2 describing all levels of response.
