# Supplementary material for: Stakeholder Attitudes Toward the Implementation of School-Based, Universal, Mental Health Screening: Student, Caregiver, and Teacher Perspectives
Source: Int J Environ Res Public Health. 2025 Dec 5;22(12):1825. doi: 10.3390/ijerph22121825 (PMC12732955; doi:10.3390/ijerph22121825)
Supplement: Supplementary file 1 [file ijerph-22-01825-s001.zip › ijerph-3969129-supplementary.pdf]

Supplementary table S1: Attitudes to screening by students in study 1 describing all levels of response.

Question/ sample	Not at all/ Definitely not	Slightly/ Probably not	Moderately/ No opinion	Very/ Probably yes	Extremely/ Definitely yes
Did you get upset when you were answering any of the questions in this whole survey?					
Full sample	70.9%	21.4%	5.8%	1.1%	0.8%
Grades 4-6 (n = 3695)	72.2%	21.7%	4.8%	0.9%	0.3%
Grades 7+ (n = 2394)	68.9%	20.9%	7.3%	1.5%	1.5%
How important do you think it is for kids to know about emotional health?					
Full sample	2.3%	4.3%	15.3%	38.7%	39.4%
Grades 4-6 (n = 3695)	2.1%	4.8%	17.6%	41.1%	34.4%
Grades 7+ (n = 2394)	2.6%	3.5%	11.8%	35.0%	47.1%
Do you think schools should check whether students have emotional health worries?					
Full sample	2.0%	4.2%	16.6%	36.9%	40.3%
Grades 4-6 (n = 3695)	1.6%	4.9%	17.7%	37.8%	38.0%
Grades 7+ (n = 2394)	2.7%	3.2%	14.8%	35.5%	43.8%
Do you think schools should teach students about emotional health?					
Full sample	1.7%	3.3%	18.0%	38.9%	38.0%
Grades 4-6 (n = 3695)	1.7%	4.0%	20.5%	42.1%	31.7%
Grades 7+ (n = 2394)	1.8%	2.2%	14.2%	34.1%	47.8%

Supplementary table S2: Attitudes to screening by educators and caregivers in study 2 describing all levels of response.

Question	Strongly Disagree	Disagree	Neither	Agree	Strongly Agree
Schools have an important role in making sure that children have good mental health					
Educators	0	0	0	15.2%	84.8%
Caregivers	0.8%	0	3.4%	34.2%	61.7%
Schools are well placed to spot the early signs that a child may be experiencing mental health difficulties					
Educators	0	5.9%	5.9%	26.5%	61.8%
Caregivers	0.8%	7.2%	6.0%	41.9%	44.2%
It is important to identify children experiencing mental health problems as early as possible					
Educators	0	0	0	5.9%	94.1%
Caregivers	0.4%	0	0	11.7%	87.9%
It is important for schools to carry out mental health checks (screening) for their students					
Educators	0	3.1%	0	53.1%	43.8%
Caregivers	0.4%	1.5%	11.9%	36.4%	49.8%
Conducting mental health checks (screening) in schools could be harmful to students					
Educators	21.2%	75.8%	24.2%	0	0
Caregivers	27.4%	47.9%	15.8%	5.8%	3.1%
If a school asks questions about mental health from students, it could put negative ideas in their head					
Educators	38.2%	29.4%	23.5%	8.8%	0
Caregivers	19.4%	45.7%	23.6%	8.9%	2.3%
I would be prepared to complete a questionnaire about (my child's /each of my students') mental health for the purpose of a routine mental health check (screening)					
Educators	3.3%	0	26.7%	43.3%	26.7%
Caregivers	0.4%	0.8%	2.3%	39.5%	57.0%
(My child/ Students at my school) is perfectly capable of honestly and accurately reporting his or her mental health					
Educators	3.0%	30.3%	33.3%	30.3%	3.0%
Caregivers	1.9%	8.0%	15.2%	53.1%	22.0%
All parents should be provided with feedback about their child's mental health following his/her participation in a mental health check (regardless of whether they are identified as having difficulties or not)					
Educators	3.0%	21.2%	21.2%	33.3%	21.2%
Caregivers	0.4%	3.4%	6.0%	38.1%	52.1%
I would be happy to work with (my child's school/ students' families) and other organizations if a mental health check showed that (my child/ student) could benefit from extra support					
Educators	0	0	5.9%	8.8%	85.3%
Caregivers	0	0.4%	0	22.3%	77.4%
	**Not at all true**	**A little true**	**Somewhat true**	**Very true**	
I plan to screen students in my school for mental health concerns in the future					
Educators	3.0%	9.1%	39.4%	48.5%	

I feel comfortable knowing how to conduct screening				
Educators	8.8%	8.8%	41.2%	41.2%
I feel comfortable knowing how to handle students identified with a mental health problem				
Educators	2.9%	5.9%	29.4%	61.8%
I am confident that the school community supports the idea of mental health screening				
Educators	0	8.8%	55.9%	35.3%

Supplementary table S3: Feedback about screening by educators who implemented school-based mental health screening in study 2 describing all levels of response.

Question	Negative/ not like my experience	Neither negative or positive/ a little like my experience	Quite positive/ Somewhat like my experience	Very positive/ very much like my experience
**Screening**				
Overall, what was your experience with screening?	0	9.1%	72.7%	18.2%
Students had trouble understanding the screening questions	9.1%	63.6%	18.2%	9.1%
Students did not take screening seriously	36.4%	27.3%	27.3%	9.1%
Students became distressed during/after screening	72.7%	18.2%	9.1%	0
Screening was difficult to fit into the regular class timetable/schedule	36.4%	27.3%	36.4%	0
Preparing for and/or conducting screening took up too much administrative time	36.4%	18.2%	36.4%	9.1%
**Feedback**				
Overall, what was your experience with following up with students who were identified through screening?	0	0	90.9%	9.1%
The screener worked well to identify students who needed help and were not previously known to school staff as needing help	0	9.1%	63.9%	27.3%
Too many students were identified who, when followed up, are not experiencing MH difficulties	36.4%	36.4%	27.3%	0
I know of a number of students who do have mental health difficulties but were not identified through screening	18.2%	45.5%	18.2%	18.2%
Caregivers for identified students appreciated our contact and the information we provided	0	18.2%	27.3%	54.5%
Caregivers supported the idea of getting their child additional mental health help when contacted by us	9.1%	9.1%	72.7%	9.1%
We did not have sufficient time/capacity to follow up identified students	54.5%	27.3%	9.1%	9.1%
There were not enough external services/resources available to support identified students	27.3%	27.3%	18.2%	27.3%
The guideline that was provided to us was helpful in providing guidance on how to follow up with identified students	0	0	63.6%	36.4%
